# Association Between Pain and Functional Disability in Patients with Shoulder Tendinopathy: A Cross-Sectional Study Using Patient-Reported and Performance-Based Measures

**DOI:** 10.3390/muscles5040070

**Published:** 2026-10-03

**Authors:** Stefanos Drimytakis, Panagiotis-Nikolaos Vlachogiorgis, Anna Christakou

**Affiliations:** 1 Department of Physiotherapy, School of Health Sciences, University of Peloponnese, 23100 Sparta, Greece; stef.drimitakis@gmail.com (S.D.); panagiothsvl@gmail.com (P.-N.V.); 2 Laboratory of Biomechanics, Department of Physiotherapy, School of Health Sciences, University of Peloponnese, 23100 Sparta, Greece

**Keywords:** SPADI, SRQ, TFAST, patient-reported outcome measures, performance-based assessment, cross-sectional study

## Abstract

Objectives: Shoulder tendinopathy is a common musculoskeletal disorder associated with persistent pain and functional disability. Although the relationship between pain and functional impairment has been investigated in other disorders, evidence in shoulder tendinopathy remains limited. Methods: This cross-sectional study included 36 patients with shoulder tendinopathy. Pain and self-reported function were assessed using the Shoulder Pain and Disability Index (SPADI); Shoulder Rating Questionnaire (SRQ); and a prespecified three-time-point Visual Analogue Scale (VAS) protocol. Affected-arm performance was evaluated using the Timed Functional Arm and Shoulder Test (TFAST). Spearman’s rank correlation coefficients (r_s_) were calculated using a Bonferroni-adjusted significance threshold of α = 0.007. Results: The SPADI Pain subscale was strongly associated with the SPADI Disability subscale (r_s_ = 0.906, *p* < 0.001), while SPADI Total was strongly and inversely associated with SRQ Total (r_s_ = −0.836, *p* < 0.001). Affected-arm TFAST performance was inversely associated with mid-test VAS pain recorded during the planned rest interval (r_s_ = −0.574, *p* < 0.001). However, TFAST performance was not significantly associated with SPADI or SRQ scores after Bonferroni correction. Conclusions: Pain and self-reported disability were significantly associated, although some strong correlations involved overlapping components of patient-reported instruments. Performance-based function was associated with pain experienced during testing but not with SPADI or SRQ scores. Patient-reported and performance-based measures may capture related but distinct dimensions of shoulder function.

## 1. Introduction

Shoulder tendinopathy is a common musculoskeletal condition associated with pain, activity limitations, and reduced functional capacity [1,2]. The rotator cuff complex plays a central role in active shoulder movement and is frequently involved in shoulder-related disorders [3]. Shoulder pain is highly prevalent in the adult population, and rotator cuff–related conditions represent an important contributor to non-traumatic shoulder symptoms [2,4]. These conditions encompass a range of clinical presentations involving the rotator cuff tendons, the long head of the biceps brachii, and other periarticular structures [3,5]. In addition to local symptoms, shoulder tendinopathy may substantially affect activities of daily living (ADL), occupational activities, physical activity, and quality of life [1,5].

The clinical presentation of shoulder tendinopathy is multifactorial and may involve interactions between tissue-related, biomechanical, psychological, and contextual factors [6,7,8]. Although tendon pathology and mechanical loading may contribute to symptom development and persistence [3,9,10], structural abnormalities do not consistently correspond to the presence or severity of pain [4,11]. This observation supports a broader clinical perspective in which pain and functional limitations are not considered to be determined exclusively by structural tissue changes. From a biopsychosocial and International Classification of Functioning, Disability and Health (ICF) perspective, pain, body functions and structures, activity limitations, participation restrictions, and contextual factors may interact and contribute to the individual’s overall functional presentation [8,12]. Accordingly, assessment of patients with shoulder tendinopathy should incorporate both symptom-related and functional outcomes [13].

Pain is one of the principal symptoms reported by individuals with rotator cuff-related shoulder disorders and may be persistent in a substantial proportion of patients [3,14,15]. Pain commonly occurs during activities involving shoulder elevation, rotation, and repetitive or sustained upper-limb loading [3,16]. Epidemiological evidence further suggests that shoulder pain is influenced by factors such as age and repetitive mechanical exposure [1,2]. However, the relationship between pain intensity and functional performance is not necessarily straightforward. Individuals with comparable structural findings may report different levels of pain and functional limitation, highlighting the importance of considering clinical outcomes from multiple dimensions rather than relying on structural findings alone [4,10,17].

Functional disability is another important component of the clinical presentation of rotator cuff–related shoulder disorders. Patients may experience difficulties with activities requiring arm elevation, load-bearing, repetitive upper-limb use, and other daily or occupational tasks [5,13,18]. These limitations may persist over time and can affect work capacity, participation in physical activity, and quality of life [1,14,19]. Importantly, functional status can be evaluated using different approaches. Patient-reported outcome measures (PROMs) provide information regarding the individual’s perceived pain and disability, whereas performance-based tests provide complementary information regarding the individual’s ability to perform specific functional tasks [13,20,21]. The integration of these approaches may therefore provide a broader characterization of the relationship between symptoms and functional status [22,23].

Despite the clinical relevance of both pain and functional disability, the relationship between these domains in patients with shoulder tendinopathy remains incompletely characterized. Previous research has examined associations between pain and disability in musculoskeletal conditions [24,25,26]; however, findings from one clinical population or measurement approach cannot necessarily be generalized to shoulder tendinopathy or to performance-based functional outcomes. In particular, examining patient-reported pain and disability alongside performance-based functional assessment may provide complementary information regarding how symptom intensity relates to different dimensions of functional status [13,21,27]. Furthermore, correlations between components of the same multidimensional questionnaire should be distinguished from associations between conceptually distinct clinical measures, particularly when interpreting patient-reported shoulder outcomes [20,28,29].

Therefore, the present study aimed to investigate the association between pain intensity and functional status in patients with shoulder tendinopathy using a multidimensional assessment framework that included patient-reported outcome measures (PROMs) and a performance-based functional assessment. The primary analysis objective was to examine the association between pain intensity and functional disability, while associations involving the different assessment modalities were considered as secondary or exploratory analyses. Based on previous evidence suggesting an association between greater shoulder pain and poorer functional status [6,25], we hypothesized that greater shoulder pain would be associated with poorer self-reported functional status. This primary hypothesis was operationalized according to the scoring direction of each instrument. Within the SPADI, higher Pain scores were expected to be positively associated with higher Disability scores. Across instruments, higher SPADI Pain scores were expected to be negatively associated with SRQ Total scores, because higher SRQ scores indicate better shoulder status. Correlations involving SPADI Total and its component subscales were considered descriptive because of the mathematical overlap between these scores and were not treated as independent evidence supporting the primary hypothesis. Associations involving SRQ Pain, affected-arm TFAST performance, and pain intensity measured in relation to the TFAST were examined as secondary exploratory analyses. VAS pain intensity was assessed at three prespecified time points: immediately before the TFAST, midway through the test during a planned rest interval, and immediately after completion of the test. The mid-test VAS assessment was used in the main correlation matrix to represent pain experienced within the functional testing context. Higher TFAST scores indicate better performance, whereas higher VAS Mid-TFAST scores indicate greater pain intensity; therefore, an inverse TFAST–VAS association would indicate that better performance was associated with lower mid-test pain. No uniform directional hypothesis was applied across all assessment measures.

## 2. Results

The study sample consisted of 36 participants with an equal gender distribution (18 men, 18 women) and a mean age of 50.89 years (SD = 13.54). Regarding symptom duration, all participants met the inclusion criteria for chronic musculoskeletal pain, reporting a persistent history of symptoms exceeding three months. The duration of the current symptomatic exacerbation varied; 36% of the participants reported an onset of their present episode within the last 15 days, while 22% reported symptoms persisting for the past month.

Clinical presentations were characterized by both pain and functional impairment in 88.9% of the cases. All participants underwent a standardized conservative physiotherapy rehabilitation programmeme. The highest levels of pain were reported during functional tasks. The most provocative activities were reaching for an object on a high shelf (M = 7.25, SD = 2.47) and sleeping on their affected side (M = 6.83, SD = 2.74). Consistent with the high pain reported during overhead reaching, functional impairment was most pronounced in similar tasks, such as placing an object on a high shelf (M = 7.05, SD = 2.60). Additionally, significant difficulty was recorded in load-bearing activities, specifically lifting a weight of approximately 4.5 kg (M = 7.13, SD = 2.53). The demographic characteristics of the participants, as well as the variables of the questionnaires and the clinical test, are presented in Table 1.

The demographic characteristics of the participants are presented in Table 2.

Table 3 presents Spearman’s rank correlations among the seven study variables. Using the Bonferroni-adjusted significance threshold of *p* < 0.007, SPADI Pain was positively associated with SPADI Disability (r_s_ = 0.906, *p* < 0.001) and negatively associated with SRQ Total (r_s_ = −0.777, *p* < 0.001). SPADI Disability and SPADI Total were also strongly and inversely associated with SRQ Total (r_s_ = −0.873 and r_s_ = −0.836, respectively; both *p* < 0.001).

VAS Mid-TFAST pain intensity was positively associated with SPADI Pain (r_s_ = 0.531, *p* < 0.001) and negatively associated with SRQ Pain (r_s_ = −0.503, *p* = 0.002). Affected-arm TFAST performance was inversely associated with VAS Mid-TFAST pain intensity (r_s_ = −0.574, *p* < 0.001), indicating that better performance was associated with lower pain intensity recorded midway through the testing sequence. In contrast, affected-arm TFAST performance was not significantly associated with SPADI Pain, SPADI Disability, SPADI Total, SRQ Pain, or SRQ Total after Bonferroni adjustment (all *p* ≥ 0.021). The remaining correlations were considered secondary or exploratory. Correlations involving SPADI Total and its constituent subscales were interpreted cautiously because of their mathematical overlap and were not considered independent evidence.

The characteristics of the SPADI variables are presented in Table 4.

The characteristics of the SRQ variables are presented in Table 5.

## 3. Discussion

The present study examined the association between pain and functional status in patients with shoulder tendinopathy using patient-reported and performance-based measures. The findings were consistent with the primary hypothesis for the self-reported outcomes. Higher SPADI Pain scores were strongly associated with higher SPADI Disability scores and with lower SRQ Total scores, consistent with the opposite scoring directions of the SPADI and SRQ. These findings indicate that self-reported pain and disability covaried within the present sample; however, the magnitude of some associations should be interpreted in light of the shared content and scoring structure of the patient-reported instruments. In the analyses involving the performance-based assessment, affected-arm TFAST performance was not significantly associated with any SPADI or SRQ score after application of the Bonferroni-adjusted threshold. However, affected-arm TFAST performance was inversely associated with VAS Mid-TFAST pain intensity. This VAS rating was obtained during the planned rest interval midway through the TFAST, immediately after completion of the first Wall Wash direction. Therefore, it reflected pain experienced within the same functional testing context, but not pain reported while the participant was actively performing the movement. The findings indicate a context-specific association between TFAST performance and mid-test pain intensity, whereas evidence of convergence between TFAST performance and the broader patient-reported outcomes was not statistically significant. The nonsignificant TFAST–PROM correlations should not be interpreted as evidence of an absence of association, particularly because the study was powered primarily to detect large effects. Furthermore, because of the cross-sectional design, the observed associations cannot establish causality, temporal direction, or mediation.

The strong correlations among the patient-reported measures require careful interpretation. Due to the fact that SPADI Total is calculated from the SPADI Pain and Disability subscales, its associations with these component scores are part–whole correlations and should not be interpreted as independent clinical findings. Although the association between SPADI Pain and SPADI Disability was strong, both scores were obtained from the same questionnaire and may partly reflect shared instrument content and common response characteristics. However, evaluating the Pain and Disability subscales as distinct dimensions is well-supported in measurement literature, which confirms their structural validity, factor stability, and independent associations with clinical constructs [28,30]. In contrast, the inverse associations between SRQ Total and the SPADI scores represent cross-instrument relationships. Their negative direction is consistent with the opposite scoring systems of the instruments, because higher SPADI scores indicate worse pain or disability, whereas higher SRQ scores indicate better shoulder status. Nevertheless, because both instruments are patient-reported, these associations demonstrate convergence in perceived shoulder status but do not establish corresponding impairment in performance-based functional capacity.

Previous study provides relevant context for the strong associations observed among the patient-reported outcomes. The validation study of the Greek SRQ reported strong concurrent validity with the SPADI in adults with rotator cuff-related shoulder pain [31], whereas SPADI validation research supports pain and disability as related but distinguishable dimensions of the instrument [28]. Accordingly, the inverse associations between SRQ Total and the SPADI scores in the present study are consistent with the instruments capturing overlapping aspects of perceived shoulder symptoms and activity limitations, while using opposite scoring directions. However, because both instruments were completed by the same participants during the same assessment session and contain related symptom and function content, shared method and content may have contributed to the magnitude of the observed correlations. These findings therefore support convergence within the patient-reported domain, but they do not establish that the instruments are interchangeable or that self-reported disability corresponds directly to performance-based functional capacity.

The absence of statistically significant associations between affected-arm TFAST performance and the SPADI or SRQ scores does not support close convergence between performance-based function and self-reported shoulder status in this sample. The TFAST was developed to assess upper-limb functional performance through standardized, time-limited tasks [27]. In a larger multisite cohort of 104 symptomatic patients, Safford et al. reported small-to-moderate correlations, ranging from 0.26 to 0.48, between TFAST performance and patient-reported shoulder status [32]. The present findings are therefore not necessarily inconsistent with previous measurement research; differences in sample size, patient characteristics, self-report instruments, and statistical thresholds limit direct comparison. Notably, affected-arm TFAST performance was significantly associated with VAS Mid-TFAST pain intensity, which was recorded during the planned rest interval immediately after completion of the first Wall Wash direction. This finding indicates a context-specific association between functional performance and pain experienced within the same testing sequence, but it does not establish that pain caused poorer performance or that this association was statistically stronger than the TFAST–PROM associations. Overall, the findings support interpreting PROMs and the TFAST as complementary rather than interchangeable sources of information about shoulder status.

Previous research indicates that the association between shoulder pain and self-reported disability may be influenced by the broader clinical and psychosocial context [6,7,33]. In a multicentre longitudinal cohort of 1030 patients receiving physiotherapy for musculoskeletal shoulder pain, Chester et al. found that lower baseline disability, greater expectations of recovery, higher pain self-efficacy, and lower pain severity at rest were associated with better patient-reported outcomes at six weeks and six months [6]. Similarly, in a cross-sectional study of 142 patients with nonoperative unilateral shoulder disorders, Lentz et al. reported that symptom duration, pain intensity, shoulder flexion range of motion, and pain-related fear collectively explained 33% of the variance in SPADI function scores [25]. These studies used substantially larger samples and multivariable analyses, whereas the present study examined bivariate correlations in 36 patients diagnosed with shoulder tendinopathy. Therefore, although the observed association between SPADI Pain and SPADI Disability is consistent with the broader literature, the present findings cannot determine the independent contribution of pain, physical impairment, or psychosocial characteristics. Instead, they provide more limited evidence that pain and perceived disability coexist within the patient-reported domain.

The item-level findings provide additional clinical context for the correlations among the patient-reported measures. Within the SPADI, the greatest mean difficulties involved lifting an object weighing approximately 4.5 kg and placing an object on a high shelf, while high pain ratings were reported during overhead reaching and when lying on the affected side. The SRQ responses showed a comparable pattern, with substantial limitations reported for overhead reaching, washing the back, combing the hair, and carrying groceries. This agreement across individual items from two different questionnaires suggests that the associations between their overall scores were also reflected in specific daily activities involving arm elevation, load handling, and self-care. These patterns are consistent with the clinical presentation of rotator cuff-related shoulder pain, in which symptoms are commonly reported during shoulder elevation and loading activities [3]. Sleep disturbance was also frequently reported in the present sample. Similarly, Cho et al. found a high prevalence of sleep disturbance among 130 patients with shoulder pain lasting at least three months and reported associations between sleep quality, pain, and self-reported shoulder function [34]. However, because the item-level findings in the present study were descriptive, they cannot determine whether sleep disturbance contributed to pain and disability or occurred as a consequence of the shoulder symptoms.

Cross-sectional and longitudinal evidence further suggests that physical impairment and patient-reported disability do not necessarily correspond closely. In a cross-sectional study of 67 non-athletic individuals with rotator cuff-related shoulder pain, Maestroni et al. identified external-rotator strength deficits in the symptomatic shoulder, but these deficits were not significantly associated with pain intensity or self-reported disability [35]. Complementing these findings, Şahinoğlu et al. retrospectively examined 39 patients with unilateral rotator cuff disease following a six-week physiotherapy program and reported only weak associations between changes in shoulder range of motion or strength and changes in SPADI scores, while most physical and patient-reported changes were not significantly associated [36]. Similarly, Alfaya et al. examined shoulder proprioception in relation to pain intensity and functional disability in individuals with subacromial impingement syndrome, further supporting the distinction between physical impairment and patient-reported outcomes [37]. Although these studies did not use the TFAST and their physical assessments differed from the performance-based measure used in the present study, they provide relevant comparative evidence that objectively measured impairment and patient-reported shoulder status may diverge. The nonsignificant TFAST–PROM associations observed in the present study are compatible with this interpretation, although they do not demonstrate an absence of association. Accordingly, PROMs, task-evoked pain, and performance-based assessments should be interpreted as complementary rather than interchangeable sources of information. Given the cross-sectional design and limited sample size, the present findings remain exploratory and cannot establish causality, temporal direction, or recovery trajectories.

### 3.1. Strengths and Limitations

A strength of the present study was the inclusion of two cross-culturally validated Greek-language patient-reported outcome measures alongside a performance-based assessment, allowing self-reported and task-based dimensions of shoulder status to be examined within the same sample. The SPADI and SRQ captured participants’ perceptions of pain and activity limitations, whereas the TFAST provided a standardized assessment of upper-limb task performance. The use of TFAST data from the clinically symptomatic limb, rather than automatically selecting the right or left limb, improved the clinical alignment between performance and symptom-related measures. All assessments were conducted before treatment by the same trained examiner under standardized clinical conditions, thereby enhancing procedural consistency and avoiding inter-examiner variation. The use of two-tailed Spearman’s rank correlations was appropriate for the characteristics of the study variables. Furthermore, the analysis distinguished a defined family of seven hypothesis-relevant comparisons, to which the Bonferroni-adjusted threshold was applied, from the remaining pairwise correlations, which were reported as secondary or exploratory. The interpretation also distinguished cross-instrument associations from mathematically overlapping SPADI components–total correlations.

Several limitations should be considered. The cross-sectional design does not permit conclusions regarding causality, temporal direction, or mediation. Although the sample of 36 participants exceeded the minimum required to detect a large effect size, it did not ensure adequate power for moderate associations. Therefore, the nonsignificant TFAST–PROM correlations should be interpreted cautiously. Convenience sampling from physiotherapy clinics within a single geographical region also limits the generalizability of the findings. Since the SPADI and SRQ assess related aspects of shoulder status through patient self-report, their associations should primarily be interpreted as evidence of convergence in participants perceived pain and function. Associations involving SPADI Total and its subscales were interpreted descriptively because these scores are structurally related. Furthermore, the bivariate analyses did not account for potentially relevant clinical or psychosocial factors (e.g., kinesiophobia, self-efficacy) or biomechanical metrics (e.g., dynamometry or range of motion). The standardized order of the functional tasks enhanced procedural consistency, although possible order or fatigue effects could not be examined separately. Similarly, the three standardized VAS assessments allowed pain intensity to be characterized before, midway through, and after the TFAST, but were not intended to provide continuous monitoring during every functional task. Nevertheless, the standardized assessment procedure and inclusion of both patient-reported and performance-based measures provide useful exploratory evidence regarding pain–function associations in this population.

### 3.2. Future Recommendations

Future research should examine these associations in larger, prospectively recruited, multicentre cohorts to improve statistical precision and external validity. Evidence syntheses and clinical studies of exercise-based and digitally delivered rehabilitation also provide practical models for repeated outcome assessment in tendinopathy and chronic shoulder pain [38,39,40]. Sample-size calculations should be based on the smallest correlation considered clinically relevant and should account for the planned adjustment for multiple comparisons. Longitudinal studies incorporating repeated measurements of SPADI, SRQ, TFAST performance, and task-evoked pain are needed to determine whether changes in self-reported symptoms and functional performance occur in parallel over time. Multivariable regression or ROC-based analyses should also examine the independent contributions of demographic characteristics, symptom duration, comorbidities, shoulder range of motion and strength, and relevant psychosocial factors, including pain-related fear, catastrophizing, recovery expectations, and self-efficacy. Future protocols should prespecify the procedure used to select the affected limb in participants with bilateral symptoms and report correlation estimates with confidence intervals. Additional measurement research should evaluate the reliability, responsiveness, and clinical relevance of affected-arm TFAST performance specifically in patients with shoulder tendinopathy and determine whether pain reported during testing provides information beyond that obtained from broader patient-reported outcome measures. These approaches would clarify the extent to which PROMs, task-evoked pain, and performance-based assessments provide complementary information about shoulder status.

## 4. Materials and Methods

### 4.1. Design

This was a cross-sectional observational study. This study has been registered and approved by the Ethics Committee of the School of Health Studies of the University (Protocol number: 13551, Date: 20 May 2025).

### 4.2. Participants

**Figure 1:**
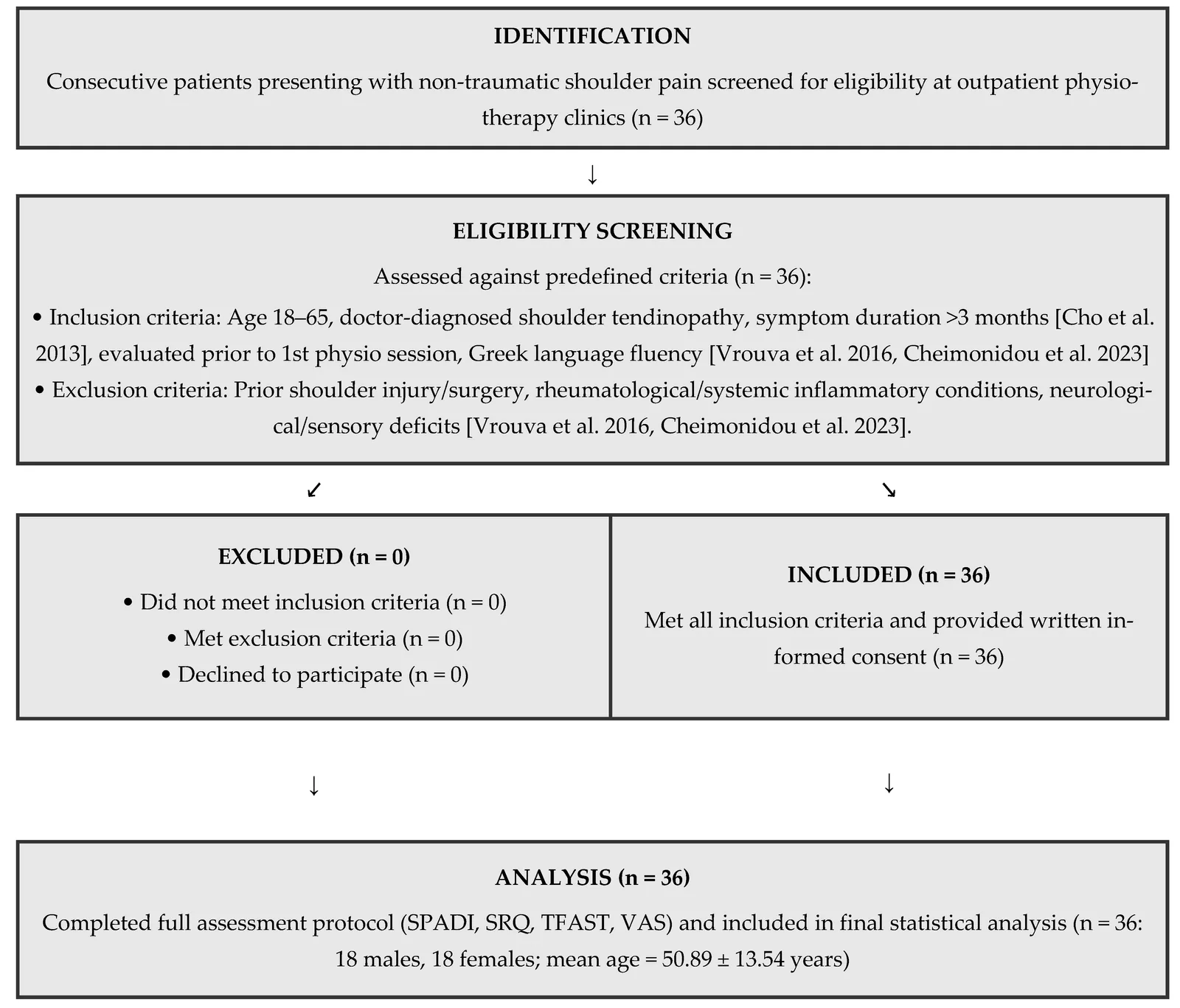
Participant recruitment and screening flowchart following STROBE guidelines [16,31,34,41].

A prior power analysis was conducted using G*Power version 3.1.9.7 for a two-tailed bivariate correlation. The analysis indicated that a minimum sample of 29 participants was required to detect a large correlation (r = 0.50) with 80% statistical power and a significance level of α = 0.05. This calculation did not account for the subsequent Bonferroni-adjusted significance threshold of α = 0.007 and was not designed to ensure adequate power for detecting moderate correlations. The final sample comprised 36 patients with shoulder tendinopathy. All participants provided written informed consent, and the study was conducted in accordance with the principles of the Declaration of Helsinki. Figure 1 shows the participants flowchart which presents the screening process with the inclusion and exclusion criteria applied in the study.

### 4.3. Instruments

(a) Shoulder Pain and Disability Index (SPADI) [41,42].

SPADI is a robust and validated instrument specifically designed to quantify pain intensity and functional impairment in patients with shoulder pathologies. The index comprises 13 items, partitioned into two distinct subscales: the first five items assess pain severity, while the subsequent eight items evaluate functional disability. Each item is scored on a 0–10 numerical scale, where 0 indicates “no pain/difficulty” and 10 represents “maximal pain/difficulty”. The SPADI’s pain subscale requires patients to rate the severity of their pain in five distinct scenarios: at its worst, when lying on the involved side, reaching for something on a high shelf, touching the back of the neck, and pushing with the involved arm. The disability subscale assesses difficulty in eight activities: washing hair, washing the back, putting on an undershirt or pullover, putting on a front-buttoning shirt, putting on pants, placing an object on a high shelf, carrying a heavy object (10 pounds), and removing something from a back pocket. The scoring procedure involves calculating the mean score for each subscale, which is then converted into a percentage (percentage score = mean × 10). The Total SPADI score is derived from the average of these two subscale percentages.

Regarding its psychometric properties, the SPADI demonstrates excellent internal consistency, with Cronbach’s alpha values of 0.92 for the pain subscale and 0.94 for the functional factor. Furthermore, its test–retest reliability is considered robust, with an Intraclass Correlation Coefficient (ICC) exceeding 0.89, indicating high measurement stability in clinically stable patients. Pearson correlation coefficients consistently range between 0.7 and 0.9, a level classified as “strong to very strong” according to Rowntree’s criteria [28,30].

(b) Shoulder Rating Questionnaire (SRQ) [31,43].

SRQ is a comprehensive, 21-item self-administered instrument designed to evaluate shoulder status across six distinct domains: global assessment, pain intensity, daily activities, recreational and athletic participation, work performance, and patient satisfaction. The global assessment component utilizes a 10 cm Visual Analogue Scale (VAS) to capture the patient’s overall perception of their shoulder condition. The scoring system employs weighted coefficients for each domain, resulting in a total score ranging from 17 to 100. Within this framework, higher numerical values indicate superior functional capacity and diminished pain levels. The Greek version of the SRQ was cross-culturally adapted and validated, specifically in patients presenting with rotator cuff tendinopathy [31]. The psychometric evaluation of the Greek SRQ demonstrated exceptional internal consistency, with a Cronbach’s alpha coefficient of 0.99, and high test–retest reliability (ICC = 0.976). Furthermore, the instrument’s concurrent validity was established through a strong correlation with the SPADI index (Pearson’s r > 0.90), confirming its construct validity and clinical utility for assessing functional outcomes in the Greek-speaking population.

(c) Timed Functional Arm and Shoulder Test (TFAST) [27].

TFAST is a performance-based assessment tool comprising three distinct, time-limited tasks. Participants are instructed to execute the maximum number of repetitions possible within a designated timeframe, focusing on key functional dimensions: range of motion (ROM), muscular endurance, and strength. The standardized equipment required for the administration of the TFAST includes low-friction cloths to facilitate wall-gliding movements, a one-gallon pitcher filled with water, and standardized cabinetry or portable tables.

In the present study, the total TFAST score was calculated using the original scoring procedure described by Shah et al. [27]. Specifically, the score was calculated as follows: TFAST total score = HHB repetitions + [(inward Wall Wash repetitions + outward Wall Wash repetitions)/4] + Gallon-Jug Lift repetitions. Division of the combined Wall Wash repetitions by four converted the two 60 s Wall Wash components into a single 30 s-equivalent score and prevented this task from being disproportionately weighted in the total score. Shah et al. reported reliability coefficients ranging from 0.80 to 0.94 for the individual TFAST tasks and an ICC of 0.83 for the total score in the symptomatic group [27]. The reported Standard Error of Measurement was 6.7 repetitions, and the Minimal Detectable Change at the 95% confidence level (MDC95) was 18.5 repetitions [27]. In a subsequent study involving patients with shoulder problems, Safford et al. reported small-to-moderate associations (r = 0.26–0.48) between TFAST performance and patient-reported shoulder status, together with evidence supporting the reliability, validity, and responsiveness of the instrument [32]. These findings support the use of the TFAST as a performance-based measure of upper-limb function, while also indicating that it should not be considered interchangeable with patient-reported measures of pain and disability.

(d) Visual Analogue Scale (VAS) [44].

The Visual Analogue Scale (VAS) is a widely used, unidimensional instrument designed to assess subjective pain intensity. It consists of a 10 cm horizontal line anchored by “no pain” at the left endpoint and “worst imaginable pain” at the right endpoint. Participants were instructed to place a mark on the line corresponding to their current pain intensity in the clinically affected shoulder. The distance from the left endpoint to the participant’s mark was measured in centimetres and recorded on a scale from 0 to 10, with higher scores indicating greater pain intensity. In the present study, the VAS was administered at three prespecified time points in relation to the TFAST: before the test (VAS Pre-TFAST), midway through the test (VAS Mid-TFAST), and immediately after the test (VAS Post-TFAST). The detailed timing of these measurements is described in the Procedure section. Previous evidence supports the reliability and validity of the VAS for the assessment of pain intensity in adults [44,45]. Its simplicity, rapid administration, and sensitivity to changes in pain intensity make it suitable for repeated measurements within a functional testing protocol. Comparisons with other pain-assessment instruments, including the Numerical Rating Scale, support its validity, although scores obtained from different types of pain scales should not be considered directly interchangeable [45,46].

### 4.4. Procedure

Data collection was conducted at the physiotherapy clinics during the patients’ initial physiotherapy session, prior to the initiation of any therapeutic intervention. All assessments were performed on-site in a controlled clinical setting, with only the examiner and the participant present throughout the evaluation process. Eligible participants were identified based on the predefined inclusion and exclusion criteria. Prior to participation, all individuals received detailed verbal and written information regarding the study procedures. Written informed consent was obtained from each participant. Participants were informed of their right to withdraw from the study at any time without consequence and were assured that all collected data would remain anonymous in any subsequent publication.

Data collection was completed during a single session. Participants first completed a demographic data form, followed by the self-administered questionnaires, namely the Shoulder Pain and Disability Index (SPADI) and the Shoulder Rating Questionnaire (SRQ). Standardized instructions were provided before questionnaire completion, and clarification was offered when necessary to ensure accurate understanding. All questionnaires were completed independently within the physiotherapy facility.

Upon completion of the self-reported measures, participants underwent the Timed Functional Arm and Shoulder Test (TFAST), which consisted of three subtests: Head to Hand and Back (HHB), Wall Wash, and Gallon-Jug Lift. Prior to testing, standardized verbal instructions were provided to ensure full comprehension of the procedures. Participants were instructed to perform each task as quickly and safely as possible, without intentionally provoking pain, and to complete the maximum number of repetitions within the allocated time.

The HHB and Gallon-Jug Lift subtests were each performed for 30 s, whereas the Wall Wash subtest was performed for 60 s in each rotational direction. A standardized 30 s rest interval was provided between subtests to minimize fatigue effects. Participants were informed that short pauses were permitted if required; however, timing continued during any rest periods. If a participant was unable to perform a specific subtest, a score of zero was assigned.

Pain intensity was assessed using the VAS at three time points that had been prespecified before data collection. The first measurement was obtained immediately before initiation of the TFAST (VAS Pre-TFAST). The second measurement was obtained midway through the TFAST, during the planned rest interval between the two Wall Wash directions and immediately after completion of the first direction (VAS Mid-TFAST). The third measurement was obtained immediately after completion of the entire TFAST (VAS Post-TFAST). The mid-test measurement was therefore completed during a planned rest interval and not while the participant was actively performing the Wall Wash movement. At all three time points, participants were instructed to report their current pain intensity in the clinically affected shoulder using the same 10 cm VAS.

In participants with unilateral symptoms, testing was initiated with the asymptomatic upper limb, followed by the symptomatic limb. In participants with bilateral symptoms (n = 3), the limb associated with lower self-reported pain intensity was assessed first, followed by the more painful limb. For all correlation analyses involving TFAST, the symptomatic limb was used in unilateral cases, whereas the more painful limb, based on the participant’s self-reported pain intensity, was designated as the index limb in bilateral cases. The VAS Mid-TFAST measurement used in the correlation analyses referred to pain intensity in the same symptomatic or index limb. For each subtest, the total number of repetitions was ordered. To ensure equal weighting across components within a standardized 30 s framework, repetitions from the Wall Wash task (internal and external rotation combined) were summed and divided by four, thereby yielding a 30 s-equivalent score, following the standardized scoring procedure outlined by Shah et al. [27]. The overall TFAST score was calculated as the aggregate of the standardized subtest scores.

All assessments were performed by a single trained evaluator to ensure procedural consistency. No additional verbal encouragement beyond the standardized instructions was provided during testing.

### 4.5. Statistical Analysis

Descriptive statistics, including means, standard deviations, skewness, and kurtosis, were calculated to summarize the demographic and clinical variables. The Kolmogorov–Smirnov test was used to assess the distributions of the continuous variables. Because the analysis included ordinal questionnaire scores and variables that did not consistently satisfy the assumptions of parametric analysis, associations were examined using Spearman’s rank correlation coefficient (r_s_). All correlation tests were two-tailed.

The main correlation matrix included seven variables: SPADI Pain, SPADI Disability, SPADI Total, SRQ Pain, SRQ Total, affected-arm TFAST performance, and VAS Mid-TFAST pain intensity. Although the VAS assessment protocol included three prespecified measurement time points, VAS Mid-TFAST was included in the main correlation matrix because it represented pain intensity recorded within the functional testing sequence. VAS Pre-TFAST and VAS Post-TFAST were retained as additional descriptive measures of pain intensity before and immediately after the functional test, respectively. The Bonferroni-adjusted inferential family comprised seven hypothesis-relevant correlations: SPADI Pain with SPADI Disability, SPADI Pain with SRQ Total, SPADI Disability with SRQ Total, SPADI Total with SRQ Total, SPADI Pain with VAS Mid-TFAST, SRQ Pain with VAS Mid-TFAST, and affected-arm TFAST with VAS Mid-TFAST. To account for multiple testing within this family, the significance threshold was set at α = 0.007 (0.05/7). Correlations with *p* < 0.007 were considered statistically significant, whereas correlations with *p* ≥ 0.007 were considered not statistically significant.

The remaining pairwise correlations in the complete matrix were reported for completeness and treated as secondary or exploratory. These correlations were interpreted cautiously and were not considered independent confirmatory evidence, particularly when they involved mathematically overlapping questionnaire components, such as SPADI Total and its constituent subscales. Statistical analyses were performed using IBM SPSS Statistics, version 31.0.

## 5. Conclusions

In patients with shoulder tendinopathy, the strong associations between self-reported pain and disability were consistent with the primary hypothesis and demonstrated convergence in perceived shoulder status, although shared questionnaire content should be considered when interpreting their magnitude. Affected-arm TFAST performance was associated with VAS Mid-TFAST pain intensity recorded during the planned rest interval within the functional testing sequence, while its associations with the broader SPADI and SRQ scores did not reach the Bonferroni-adjusted significance threshold. This pattern highlights the context-specific nature of the pain–function relationship and suggests that patient-reported outcomes, task-evoked pain, and performance-based assessments provide complementary rather than interchangeable information about shoulder status. By examining these assessment domains within the same clinical sample, the present study contributes to a more differentiated understanding of pain and function in shoulder tendinopathy. The cross-sectional design and characteristics of the sample warrant appropriate caution when generalizing the findings, while larger prospective studies may further clarify their clinical relevance and potential value for outcome prediction.

## Figures and Tables

**Table 1:** Descriptive statistics of the sample.

Characteristics	Minimum Value	Maximum Value	Mean ± SD	Skewness	Kurtosis
Age (years)	21	65	50.89 (13.54)	−0.77	−0.43
Weight (kg)	53	107	76.81 (13.89)	0.26	−0.90
Height (cm)	153	190	170.22 (9.58)	0.04	−0.97
Years of employment	3	45	24.50 (11.88)	−0.13	−0.72
SPADI Total Pain Score	14	100	68.50 (23.47)	−0.97	−0.02
SPADI Total Disability Score	4	98	59.06 (25.08)	−0.54	−0.48
SPADI Total (%)	9	96	64.08 (23.69)	−0.81	−0.19
SRQ Total Score (0–100)	20	86	45.19 (16.33)	0.62	0.16
TFAST Total Score (Right limb)	22	127	55.92 (26.38)	1.08	0.74
TFAST Total Score (Left limb)	22	147	64.86 (29.67)	0.89	0.56

**Table 2:** Frequencies of the demographic data of the sample.

Characteristics		Frequency (%)
Sex	Male Female	18 (50) 18 (50)
Family Status	Married Widowed Single	28 (77.8) 3 (8.3) 3 (11.1)
Occupation	Public employee Farmer Retired Self-employed Household Gym trainer Student Private employee Musician Administrative support	6 (16.7) 2 (5.6) 9 (25) 3 (8.3) 7 (19.4) 1 (2.8) 2 (5.6) 3 (8.3) 2 (5.6) 1 (2.8)
Sedentary Work	Yes No	6 (16.7) 30 (83.3)
Sports Participation	Yes No	6 (16.7) 30 (83.3)
Doctor visit	Yes No	36 (100) 0 (0)
Conservative Treatment	Yes No	36 (100) 0 (0)
History of Physiotherapy	Yes No	32 (88.9) 4 (11.1)
Medication Treatment for Symptoms	Yes No	32 (88.9) 4 (11.1)
Frequency of Medication Use	None Daily Weekly Rarely	4 (11.1) 24 (66.7) 6 (16.7) 2 (5.6)
Symptoms	Pain Functional limitations Both	4 (11.1) 0 (0) 32 (88.9)

**Table 3:** Correlations between variables of the study.

	SPADI Pain	SPADI Disability	SPADI Total	SRQ Pain	SRQ Total	TFAST Affected Arm	VAS Mid-TFAST
SPADI Pain	1						
SPADI Disability	0.906 *	1					
SPADI Total	0.959	0.977	1				
SRQ Pain	−0.521	−0.552	−0.544	1			
SRQ Total	−0.777 *	−0.873 *	−0.836 *	0.716	1		
TFAST Affected Arm	−0.380	−0.385	−0.372	0.318	0.355	1	
VAS Mid-TFAST	0.531 *	0.500	0.521	−0.503 *	−0.535	−0.574 *	1

SPADI Pain: SPADI Pain Subscale Score; SPADI Disability: SPADI Disability Subscale Score; SPADI Total: SPADI Total Score; SRQ Pain: SRQ Pain Domain Score; SRQ Total: SRQ Total Score; TFAST Affected Arm: TFAST performance score for the clinically affected upper limb; VAS Mid-TFAST: pain intensity reported in the clinically affected upper limb midway through the TFAST, during the planned rest interval immediately after completion of the first Wall Wash direction. Values are Spearman’s rank correlation coefficients (r_s_). Coefficients marked with an asterisk have *p* < 0.007. The Bonferroni-adjusted threshold was applied to the seven hypothesis-relevant comparisons defined in the Statistical Analysis section; all remaining pairwise correlations were considered secondary or exploratory.

**Table 4:** Characteristics of the SPADI Variables.

Variable	Minimum Value	Maximum Value	Mean ± SD	Skewness	Kurtosis
Worst Pain Intensity (0–10)	2.00	10.00	7.58 ± 1.70	−1.16	2.04
Pain when lying on affected side (0–10)	0.00	10.00	6.83 ± 2.74	−1.28	0.47
Pain when reaching high self (0–10)	1.00	10.00	7.25 ± 2.47	−1.07	0.34
Pain when touching back of neck (0–10)	0.00	10.00	6.58 ± 3.00	−0.83	−0.60
Pain when pushing with affected limb	1.00	10.00	6.67 ± 2.51	−0.93	−0.02
Difficulty washing hair (0–10)	0.00	10.00	6.56 ± 2.96	−0.75	−0.37
Difficulty washing back (0–10)	0.00	10.00	6.83 ± 2.71	−0.95	0.06
Difficulty putting on t-shirt	0.00	10.00	6.11 ± 2.42	−0.90	0.36
Difficulty wearing buttoned shirt (0–10)	0.00	9.00	5.31 ± 2.93	−0.49	−1.28
Difficulty putting on pants (0–10)	0.00	9.00	4.22 ± 2.82	−0.09	−1.17
Difficulty placing object on high self (0–10)	1.00	10.00	7.06 ± 2.61	−1.06	0.41
Difficulty lifting~4.5 kg (0–10)	0.00	10.00	7.14 ± 2.53	−1.13	1.19
Difficulty reaching back pocket (0–10)	0.00	10.00	6.47 ± 3.31	−0.83	−0.89

**Table 5:** Characteristics of the SRQ Variables.

Characteristics	Answers	Frequency (%)
Dominant arm	Right	31 (86.1)
Dominant arm	Left	4 (11.1)
Dominant arm	Both	1 (2.8)
Affected arm (evaluated/treated)	Right	24 (66.7)
Affected arm (evaluated/treated)	Left	9 (25.0)
Affected arm (evaluated/treated)	Both	3 (8.3)
Pain during activity (last month)	Very severe	8 (22.2)
Pain during activity (last month)	Severe	18 (50.0)
Pain during activity (last month)	Moderate	9 (25.0)
Pain during activity (last month)	Mild	1 (2.8)
Pain during activity (last month)	None	0 (0.0)
Pain at rest (last month)	Very severe	6 (16.7)
Pain at rest (last month)	Severe	17 (47.2)
Pain at rest (last month)	Moderate	6 (16.7)
Pain at rest (last month)	Mild	5 (13.9)
Pain at rest (last month)	None	2 (5.6)
Sleep disturbance due to shoulder	Every day	10 (27.8)
Sleep disturbance due to shoulder	Several days/week	20 (55.6)
Sleep disturbance due to shoulder	Once/week	3 (8.3)
Sleep disturbance due to shoulder	<1 day/week	1 (2.8)
Sleep disturbance due to shoulder	Never	2 (5.6)
Frequency of severe pain	Every day	13 (36.1)
Frequency of severe pain	Several days/week	18 (50.0)
Frequency of severe pain	Once/week	3 (8.3)
Frequency of severe pain	<1 day/week	1 (2.8)
Frequency of severe pain	Never	1 (2.8)
ADL shoulder function	Severe limitation/disability	9 (25.0)
ADL shoulder function	Severe limitation	15 (41.7)
ADL shoulder function	Moderate limitation	9 (25.0)
ADL shoulder function	Mild limitation	1 (2.8)
ADL shoulder function	No limitation	2 (5.6)
Difficulty dressing (shirt)	Unable	3 (8.3)
Difficulty dressing (shirt)	Severe difficulty	17 (47.2)
Difficulty dressing (shirt)	Moderate difficulty	13 (36.1)
Difficulty dressing (shirt)	Mild difficulty	0 (0.0)
Difficulty dressing (shirt)	None	3 (8.3)
Difficulty combing hair	Unable	8 (22.2)
Difficulty combing hair	Severe difficulty	17 (47.2)
Difficulty combing hair	Moderate difficulty	8 (22.2)
Difficulty combing hair	Mild difficulty	0 (0.0)
Difficulty combing hair	None	3 (8.3)
Difficulty reaching overhead	Unable	10 (27.8)
Difficulty reaching overhead	Severe difficulty	17 (47.2)
Difficulty reaching overhead	Moderate difficulty	5 (13.9)
Difficulty reaching overhead	Mild difficulty	3 (8.3)
Difficulty reaching overhead	None	1 (2.8)
Difficulty washing back	Unable	16 (44.4)
Difficulty washing back	Severe difficulty	11 (30.6)
Difficulty washing back	Moderate difficulty	4 (11.1)
Difficulty washing back	Mild difficulty	3 (8.3)
Difficulty washing back	None	2 (5.6)
Difficulty lifting groceries (3.5–4.5 kg)	Unable	14 (38.9)
Difficulty lifting groceries (3.5–4.5 kg)	Severe difficulty	11 (30.6)
Difficulty lifting groceries (3.5–4.5 kg)	Moderate difficulty	6 (16.7)
Difficulty lifting groceries (3.5–4.5 kg)	Mild difficulty	2 (5.6)
Difficulty lifting groceries (3.5–4.5 kg)	None	3 (8.3)
Function in sports/leisure	Very severe limitation/disability	5 (13.9)
Function in sports/leisure	Severe limitation	16 (44.4)
Function in sports/leisure	Moderate limitation	13 (36.1)
Function in sports/leisure	Mild limitation	2 (5.6)
Function in sports/leisure	No limitation	0 (0.0)
Preferred activity affected	None	0 (0.0)
Preferred activity affected	Light exercise (home/gym)	24 (66.7)
Preferred activity affected	Swimming	3 (8.3)
Preferred activity affected	Basketball	2 (5.6)
Preferred activity affected	Boxing	1 (2.8)
Preferred activity affected	Gym	2 (5.6)
Preferred activity affected	Golf	1 (2.8)
Preferred activity affected	Gardening	1 (2.8)
Preferred activity affected	Music (violin/piano)	1 (2.8)
Preferred activity affected	Volleyball	1 (2.8)
Work status (last month)	Employed	13 (36.1)
Work status (last month)	Household	13 (36.1)
Work status (last month)	Student	2 (5.6)
Work status (last month)	Retired	8 (22.2)
Unable to perform usual work	No response (Q15)	7 (19.4)
Unable to perform usual work	Every day	6 (16.7)
Unable to perform usual work	Several days/week	18 (50.0)
Unable to perform usual work	Once/week	2 (5.6)
Unable to perform usual work	<1 day/week	1 (2.8)
Unable to perform usual work	Never	2 (5.6)
Worked less due to shoulder	No response (Q15)	7 (19.4)
Worked less due to shoulder	Every day	11 (30.6)
Worked less due to shoulder	Several days/week	13 (36.1)
Worked less due to shoulder	Once/week	2 (5.6)
Worked less due to shoulder	<1 day/week	2 (5.6)
Worked less due to shoulder	Never	1 (2.8)
Changed work method	No response (Q15)	7 (19.4)
Changed work method	Every day	11 (30.6)
Changed work method	Several days/week	14 (38.9)
Changed work method	Once/week	2 (5.6)
Changed work method	<1 day/week	0 (0.0)
Changed work method	Never	2 (5.6)
Satisfaction with shoulder condition	Poor	23 (63.9)
Satisfaction with shoulder condition	Fair	8 (22.2)
Satisfaction with shoulder condition	Good	2 (5.6)
Satisfaction with shoulder condition	Very good	3 (8.3)
Satisfaction with shoulder condition	Excellent	0 (0.0)
Primary improvement goal	Pain	12 (33.3)
Primary improvement goal	ADL	10 (27.8)
Primary improvement goal	Sports/leisure	7 (19.4)
Primary improvement goal	Work	7 (19.4)
Secondary improvement goal	Pain	18 (50.0)
Secondary improvement goal	ADL	10 (27.8)
Secondary improvement goal	Sports/leisure	3 (8.3)
Secondary improvement goal	Work	5 (13.89)

## Data Availability

The data presented in this study are available on request from the corresponding author.
